# Randomized trial of tapas acupressure technique for weight loss maintenance

**DOI:** 10.1186/1472-6882-12-19

**Published:** 2012-03-15

**Authors:** Charles R Elder, Christina M Gullion, Lynn L DeBar, Kristine L Funk, Nangel M Lindberg, Cheryl Ritenbaugh, Gayle Meltesen, Cherri Gallison, Victor J Stevens

**Affiliations:** 1 Kaiser Permanente Center for Health Research, 3800 N. Interstate Ave., Portland, OR 97227, USA; 2 University of Arizona, Tucson, AZ, USA; 3 Healing Touch Acupuncture, Portland, OR, USA

**Keywords:** Obesity, Weight-loss maintenance, Energy medicine, Acupressure

## Abstract

**Background:**

Obesity is an urgent public health problem, yet only a few clinical trials have systematically tested the efficacy of long-term weight-loss maintenance interventions. This randomized clinical trial tested the efficacy of a novel mind and body technique for weight-loss maintenance.

**Methods:**

Participants were obese adults who had completed a six-month behavioral weight-loss program prior to randomization. Those who successfully lost weight were randomized into either an experimental weight-loss maintenance intervention, Tapas Acupressure Technique (TAT^®^), or a control intervention comprised of social-support group meetings (SS) led by professional facilitators. TAT combines self-applied light pressure to specific acupressure points accompanied by a prescribed sequence of mental steps. Participants in both maintenance conditions attended eight group sessions over six months of active weight loss maintenance intervention, followed by an additional 6 months of no intervention. The main outcome measure was change in weight from the beginning of the weight loss maintenance intervention to 12 months later. Secondary outcomes were change in depression, stress, insomnia, and quality of life. We used analysis of covariance as the primary analysis method. Missing values were replaced using multiple imputation.

**Results:**

Among 285 randomized participants, 79% were female, mean age was 56 (standard deviation (sd) = 11), mean BMI at randomization was 34 (sd = 5), and mean initial weight loss was 9.8 kg (sd = 5). In the primary outcome model, there was no significant difference in weight regain between the two arms (1.72 kg (se 0.85) weight regain for TAT and 2.96 kg (se 0.96) weight regain for SS, p < 0.097) Tests of between- arm differences for secondary outcomes were also not significant. A secondary analysis showed a significant interaction between treatment and initial weight loss (p < .036), with exploratory *post hoc *tests showing that greater initial weight loss was associated with more weight regain for SS but less weight regain for TAT.

**Conclusions:**

The primary analysis showed no significant difference in weight regain between TAT and SS, while secondary and post hoc analyses indicate direction for future research.

**Trial Registration:**

ClinicalTrials.gov: NCT00526565

## Background

Complementary and alternative medicine (CAM) approaches for obesity treatment and weight control are popular and appealing [1-3]. A variety of CAM modalities--such as acupuncture,[4] acupressure, meditation, stress reduction, and guided imagery--have been described as primary or ancillary approaches to weight reduction [1,5,6]. However, few studies have systematically tested the efficacy of these techniques [6-10]. This is the report of a randomized, controlled clinical trial of a CAM approach to weight-loss maintenance.

Obesity is an urgent public health problem, as the prevalence has increased dramatically in the past 30 years, with more than two thirds of adults in the United States now overweight or obese [11,12]. A number of behavioral and dietary interventions are effective in inducing initial weight loss [13-16], but weight regain is a common problem following virtually all dietary and behavioral weight-loss interventions [14,17-19].

Only a few clinical trials have systematically tested the efficacy of long-term weight-loss maintenance interventions [13,20]. For instance, in the recently completed Weight Loss Maintenance Trial [21], monthly participant contacts with a weight-loss counselor were shown to improve long-term maintenance of initial weight loss compared to a no-further-treatment control. Although these results are promising, more work is needed to optimize weight-loss maintenance strategies, including evaluation of CAM approaches.

In principle, training in a mind and body technique could provide a convenient and practical tool for managing stress, controlling food cravings, and maintaining healthy lifestyle habits beyond the conclusion of a weight management intervention. The term "energy psychology" refers to a family of mind and body interventions that involve tapping or holding specific acupressure points (acupoints), combined with specific mental imagery, as treatment for a wide range of medical and psychological conditions. Popular techniques include Thought Field Therapy (TFT)^® ^[22], Emotional Freedom Technique (EFT)^® ^[23] and Tapas Acupressure Technique (TAT)^® ^[24].

The efficacy of these techniques has not been adequately tested in randomized trials, but such a pursuit is potentially worthwhile for several reasons. First, numerous case reports and anecdotes suggest dramatic results across a range of medical conditions [25]. Second, information about the techniques is widely available in books and websites, which would facilitate widespread use of an effective technique. Third, energy psychology approaches can reference the ancient practice and theory of traditional Chinese medicine as an explanatory model. Chinese medicine theorizes that the free flow of energy, or Qi, through the body-mind system is essential for balance and health [26]. This flow occurs through a series of systemic energy pathways called meridians. If Qi is blocked, overall health and balance suffer. The techniques purport to treat disease and maintain health by restoring the proper flow of Qi through this meridian system. However, although "blocked Qi" is the mechanism of action for the effects of TAT purported by the technique's developer and many TAT practitioners, there are other potential pathways for its impact, including as a behavioral tool used in the moment to reduce cravings as well as to promote stress reduction and relaxation.

We previously published results of a controlled, randomized pilot study evaluating the feasibility of two CAM interventions for weight-loss maintenance, TAT and Qigong (a Chinese movement meditation) [27]. Ninety-two participants who had successfully lost at least 3.5 kg in a conventional 12-week weight-loss program were randomly assigned to one of these interventions or to a social support arm. At six months post randomization, those in the TAT arm maintained a weight loss of 1.2 kg more than the social support arm (p < .089), and 2.8 kg more than the Qigong arm (p < .001). Based on these results, we conducted a larger randomized controlled trial, the LIFE study, to determine the efficacy of TAT as a weight-loss maintenance tool. This paper presents the results from that trial.

## Methods

### Study design

**Figure 1:**
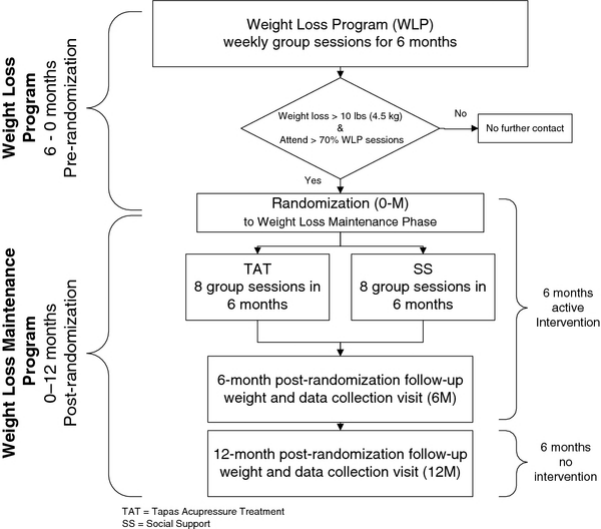
**Study Design**.

Details of the study rationale, design, and procedures of the LIFE study have already been published [28], so a brief summary is given here. The study used a two-arm randomized design. (Figure 1). Participants were obese adults who participated in an initial, six-month behavioral weight-loss program (WLP). Those who lost at least 4.54 kg (10 lb) and attended greater than 70% of the weekly WLP group sessions were eligible for randomization into one of two arms of the weight-loss maintenance phase of the trial. The experimental intervention arm consisted of instruction and application of the *Tapas Acupressure Technique *(TAT, a form of self-acupressure described below), while the control intervention arm consisted of social support-group meetings (SS). Both arms had identical group meeting schedules over about six months, totaling 13 contact hours in eight group sessions. The main outcome measure for the trial was change in weight from randomization to 12 months post randomization. Each participant was thus enrolled in the trial for 18 months: 6 month weight loss program plus 12 months weight loss maintenance phase, with the active weight loss maintenance intervention confined to the first six months of the weight loss maintenance phase.

### Participants

Participants were members of the Kaiser Permanente Northwest (KPNW) health maintenance organization who were at least 30 years old, had a BMI between 30 and 50 inclusive, weighed less than 181.8 kg (400 lb), and lived in the Portland, Oregon, metropolitan area. Medical exclusions included recent (within the previous two years) cardiovascular event, cancer treatment, or inpatient psychiatric hospitalization. People were excluded if they had diabetes and were injecting insulin, were taking weight-loss medications, or had undergone bariatric surgery or liposuction over the previous year. Additional exclusions included prior use of acupuncture or acupressure for weight management, plans to leave the area prior to the end of the study, weight loss of more than 9.1 kg (20 lb) over the prior six months, breast feeding, pregnancy, or plans to become pregnant before the end of the study. The final eligibility criterion was attendance at the first WLP group session.

### Recruitment

Two outreach strategies were implemented, both emphasizing participation in a study about weight management. One strategy was direct-letter contact to KPNW members who appeared to meet eligibility criteria based on query of the KPNW electronic medical record database. The other strategy was study publicity to KPNW members and employees via newsletters, posters, and brochures. Interested individuals contacted the study to schedule a group information session. At that session, those interested in pursuing further screening and enrollment were scheduled for an individual study entry visit where eligibility was determined and informed consent was obtained. Study entry began at the first session of the WLP.

Recruitment for the weight loss sessions began in January 2008, and continued through January 2009. All participants completed follow up by the end of August, 2010. All study materials and protocols were reviewed and approved by the KPNW Institutional Review Board.

### Weight Loss Program (WLP)

The WLP preceding randomization was based on behavioral weight-loss programs developed and refined in previous studies and shown to successfully achieve a clinically meaningful weight loss in overweight and obese adults [21,29-33]. Briefly, the WLP consisted of 22 weekly group meetings during which participants were instructed and supported to reduce calories consumed and eat a healthy low-fat diet, rich in fruits and vegetables. Additionally, participants were encouraged to keep daily records of all foods and beverages consumed and to exercise at moderate intensity most days, working up to 180 minutes per week. Those who lost at least 4.54 kg (10 lb) and attended at least 70% of the weekly WLP sessions were invited to continue into the randomized weight-loss maintenance phase.

### Randomization

Participants were randomly assigned to the two maintenance interventions in equal numbers. We balanced entry into the two treatment arms by stratifying on weight loss program (WLP) group, with permuted blocks of varying size. The randomization sequence and block size were computer generated and with the exception of the statistician and programmer, we masked all staff from block-size. For each weight loss group, the study staff were provided with a sequence of sealed envelopes, each with a concealed randomization code inside. After completion of eligibility, the staff member opened the next envelope and informed the participant of his/her intervention assignment. The randomization code was a self-adhesive label that was then put on the randomization log, which served as a record of the sequence of code assignments, and assured that codes were handed out in the correct order.

### Weight-loss maintenance interventions

We conducted 18 (9 TAT and 9 SS) weight-loss maintenance groups, each with approximately 16 participants.

#### Tapas Acupressure Technique (TAT)

**Figure 2:**
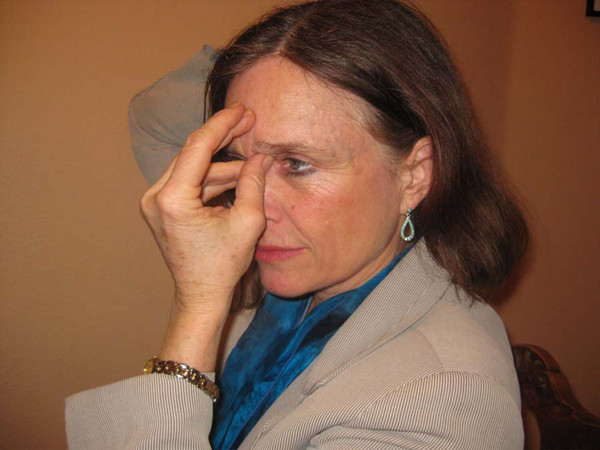
**The TAT pose**.

The TAT practice combined self acupressure with a prescribed set of mental steps. The participant was instructed to apply light touch using the tips of the thumb and fourth finger of one hand to the area 1/8- inch above the inner corner of each eye, with the middle finger of the same hand positioned on the forehead directly above the nose and about 1/2-inch above eyebrow level. The other hand was placed on the back of the head, with the palm cradling the occiput and the thumb pointing down as it rests above the hairline (Figure 2). Participants were instructed to focus on a series of statements (silent or spoken) aimed at healing and resolving barriers in a problem area, while maintaining this TAT pose. They were advised to practice TAT daily. The complete protocol took approximately 20-30 minutes; a shortened version was also taught, which could be completed in 2-5 minutes and used throughout the day as needed. Detailed participant instructions, rationale, and practitioner curriculum guides for the study protocol have been previously published [28].

A certified TAT practitioner taught the elements of its practice to participants assigned to the TAT arm. TAT certification required: 1) 60 hours of workshop attendance; 2) home study of print and audiovisual curricular materials; 3) documentation of 50 TAT sessions on self, 50 on clients, and 15 on fellow trainees; and 4) final evaluation of two TAT sessions, either live or via video, by a certified TAT trainer.

The study implemented several quality-control procedures to assure correct and uniform adherence to the TAT curriculum and protocol. First, a rating tool developed for the study was completed after each session by both the TAT facilitator and an investigator, or study staff member, who regularly observed the sessions. The session rater documented the percentage of time devoted to TAT mechanics, weight-management issues and other topics, and a description of any activities that may have deviated from the curriculum guide. TAT facilitators, the project director, and investigators participated in monthly conference calls which provided an opportunity to share experiences, address logistic concerns, and preserve consistency and adherence to the TAT treatment protocol.

In addition to practicing the TAT method during group sessions, participants also discussed personal experiences using TAT and behavioral weight management concepts such as roadblocks, limiting beliefs, and triggers. A total of three certified TAT practitioners were used in the study.

#### Social Support (SS)

The SS weight-loss maintenance arm consisted of a series of facilitated group sessions matched to the TAT intervention's meeting schedule and contact time. Participants at these sessions shared experiences and suggestions related to weight-loss maintenance strategies in a supportive environment. Sessions began with a "check in" period in which participants discussed progress and concerns. The group facilitator encouraged discussion of a weight management-related theme. Topics included roadblocks to weight-loss maintenance, emotional reactions to stress, maintaining balance, and approaches to managing food and exercise. The facilitator's role was to manage meeting logistics and guide the group discussion. No new material was introduced. A total of five SS masters-level facilitators, with specific training in support group dynamics and conducting group intervention protocols, were used in the study. Facilitators had a median of 8 (range 1-20) years of professional experience.

### Outcome measures

The primary outcome measure was change in weight from randomization (0-M) to 12 months post randomization. Participant weight was measured at 6 (6 M) and 12 (12 M) months post randomization using a calibrated scale with participants wearing indoor clothing but without shoes.

All clinical staff involved in outcome data collection were masked to participants' intervention assignment. We used procedures that are standard at this center for keeping assessment staff blind to participant treatment assignment in behavioral intervention studies. During the introductory visits, we informed participants about the importance of keeping assessment staff blinded to treatment assignment, and explained the blinding procedures. At each visit check-in, we reminded participants that assessment staff were "blinded" and asked participants to refrain from discussing any aspect of their intervention weight program or activities with assessment staff. Assessment staff wore "Shhh, I'm blinded" buttons reminding participants to refrain from discussing intervention assignment, weight, or intervention activities. Signs were posted by the scale asking participants to keep the session blinded. Data forms did not indicate assignment, and assessment staff did not have access to previous forms or data that could reveal anything about treatment assignment.

Participants provided demographic data using paper and pencil questionnaires. Study staff collected psychosocial measures (0-M, 6 M, 12 M) using previously validated assessment tools, including: *Insomnia Severity Index *(ISI) [34], a 5-item questionnaire measuring a participant's perception of his or her sleep quality in the previous two weeks; *Perceived Stress Scale *(PSS), a ten-item questionnaire with scores ranging from zero to 40 and higher scores indicating greater stress in the previous month [35,36]; *Personal Health Questionnaire-Depression Subscale *(PHQ-8), an 8-item instrument in which higher scores indicate more pervasive depression symptoms during the previous two weeks [37]; and *Quality of Life Enjoyment and Satisfaction Questionnaire-Short Form (Q-LES-Q-SF)*, used to assess quality of life [38]. We also administered a review of systems checklist inquiring about potential adverse events at each study visit.

At each weight-loss maintenance group session, participants in the TAT arm reported the average number of days each week they practiced TAT on their own.

### Statistical methods

The primary efficacy analysis was carried out in the intent-to-treat sample; that is, all randomized participants were included in the analysis. To complete missing outcome and covariate data, we carried out multiple imputation by data augmentation [39] using SAS^® ^V9.2 PROC MI.

The primary hypothesis test of efficacy measured the adjusted difference between SS and TAT, estimated by the weight change from 0-M to 12-month (12 M) follow-up (12 M - (0-M)) in an analysis of covariance (ANCOVA) with four planned covariates: change in weight from beginning of weight-loss program (entry) to randomization ((0-M)-entry), weight at 0-M, race, and sex. All weight-change measures were defined so that positive values indicated weight gain, while negative values indicated weight loss. Race was dichotomized as white vs. other. The critical values for significance were set a priori at α = .05 for main effects tests and .10 for interactions tests [40].

We also evaluated change in weight during the active weight-loss maintenance intervention (6 M-(0-M)) and four other secondary outcomes (change in stress, depression, insomnia, quality of life from 0-M to 12 M) using the same model and procedure as for the primary outcome analysis.

An assumption of ANCOVA is that the association between each covariate and the outcome is the same across treatment arms. This is evaluated in the test of the interaction between the treatment arm and that covariate [41,42]. When the slopes are not the same, the adjusted difference between treatments varies over the range of the covariate. Therefore, in a planned secondary analysis, before adding additional hypothesized covariates to the "initial" outcome model, we added the four terms for treatment-by-covariate interaction. These were sequentially stepped out in order of decreasing *p *value, until only the significant interaction terms remained, ending with the "augmented" model.

Next, we evaluated the contribution of additional covariates (education, age at entry, 0-M score on PHQ-8, ISI, and PSS, and percent attendance at sessions during the active 6-month weight loss-maintenance intervention) to prediction of weight change in the augmented model. We hypothesized that these covariates might be related to success in weight-loss maintenance at 12 M, based on previous analyses of relationships between these measures and weight loss [32,33]. Separately for each covariate, the interaction with treatment was tested first in the augmented model and, if significant, it was retained in the model. If the interaction term was not significant, it was dropped and the main effect of the covariate was estimated in the augmented model. All significant covariates (and any significant interaction terms)were then added to the model, and those that were not significant were stepped out to obtain the "final" best model reported here.

Finally, if any covariate had differing slopes between treatments (i.e., significant interaction with treatment) in the augmented model, we followed up with analyses intended to characterize the pattern of effects. To do this, we estimated the least squares means for treatment at selected values of the covariate and tested the difference between arms. These analyses were *post hoc *as they were carried out only as a follow-up if a significant interaction was found. These tests were used to establish the magnitude and direction of treatment effect at these values.

### Sample size

Starting with the pilot study estimates of difference in weight change between the TAT and Self-directed control arms at 6 M follow-up (0.1 and 1.2 kg, respectively, standard deviation (sd) = 2.3, Cohen's *d *= 0.48), we estimated the needed sample size for the primary hypothesis test of treatment effect in this larger study. The Type I error rate, α, is 2-tailed, at .05. We assumed that there might be a smaller difference between groups as a result of sampling or experimental error in the new study and that the difference between arms might diminish over the longer follow-up period of 12 months, so we modeled differences of change scores of 0.7 to 1.1 kg. Also, to account for the added error from multiple imputation, we assumed a 10% higher sd (2.53 kg) than in the pilot study, which decreased the effect size and increased the needed sample size slightly. A final sample (assuming the higher sd) of about 144 per arm at month 12 was deemed sufficient to detect an effect size of about .33-.34 (e.g., 1.20 vs. 0.36 or 1.00 vs. 0.16 kg) with a power of .80.

## Results

### Participant flow

**Figure 3:**
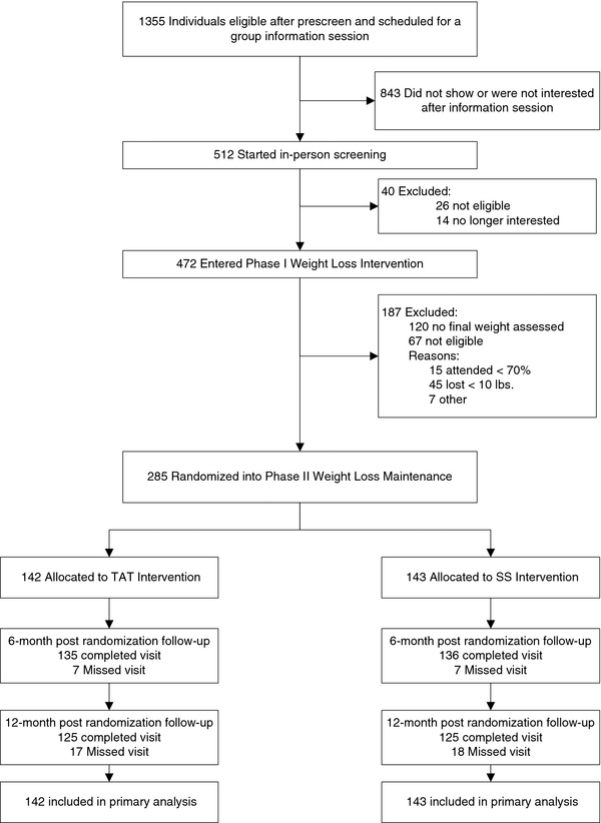
**Flow of Study Participants**.

As Figure 3 shows, of the 472 participants who enrolled in the initial weight loss program, 285 (60%) achieved weight loss and attendance targets and were randomized, with 142 assigned to TAT and 143 to SS. The TAT and SS participants attended an average of 73% and 70%, respectively, of weight-loss maintenance intervention sessions. We collected final outcome (12 M) weight on 88% of participants.

### Participant characteristics

Among the 285 randomized participants, mean age was 56.2 years (standard deviation [sd] 11.2), mean BMI at 0-M was 33.9 (sd 5.1), and mean weight loss during the WLP preceding 0-M was 9.8 kg (sd 4.6). Seventy nine percent were female. Inspection of Table 1 suggests that participants were demographically similar across study arms at the time of randomization. Minority participation, although higher in the TAT arm, was low overall, consistent with demographic patterns among HMO members in the metropolitan Portland area.

**Table 1:** Study characteristics for randomized participants

Characteristic	TAT (n = 142)	SS (n = 143)	Total (n = 285)
		*mean (s.d.) or %*	

Age, years	55.4 (11.2)	56.9 (11.2)	56.2 (11.2)

Weight @ randomization, kg	94.5 (18.1)	94.6 (15.4)	94.5 (16.8)

Weight change in WLP, kg	-9.6 (4.5)	-9.9 (4.8)	-9.8 (4.6)

BMI (@ randomization	33.9 (5.2)	33.9 (5.0)	33.9 (5.1)

BMI category @ randomization, kg			

Normal BMI < 25	0.7	0.7	0.7

Overweight (BMI ≥ 25 and < 30)	28.2	23.1	25.6

Obese Stage 1 (BMI ≥ 30 and < 35)	36.6	39.9	38.3

Obese stage 2 (BMI ≥ 35)	34.5	36.4	35.4

Women (%)	78.9	79.7	79.3

Minority participants (%)	8.5	4.3	6.4

Education, highest level (%)			

High school or less	8.5	7.7	8.1

Some college	36.6	32.9	34.7

Completed college	22.5	30.1	26.3

Post college	32.4	29.4	30.9

Household income, dollars (%)			

<$29,999	10.8	7.3	9.1

$30,000-44,999	15.2	18.9	17.1

$45,000-59,999	16.9	23.4	20.1

$60,000-74,999	22.4	16.9	19.6

$75,000-89,999	13.5	9.8	11.6

$90,000+	22.1	23.8	22.5

### Change in weight

In our primary analysis, adjusting for the initial planned covariates (Table 2, initial model), the estimated weight regain at 12 M (the primary outcome measure) was 1.72 kg (se 0.85) for TAT and 2.96 kg (se 0.96) for SS. The adjusted mean difference between the two arms (TAT-SS = - 1.24 kg (se 0.74)) was not significant (F[1,279] = 2.79, p < .098). However, we found a significant interaction between treatment and weight loss in the WLP (p < .0829), as well as an interaction just short of significance between treatment and 0-M weight (p < .1088), indicating that the treatment effect was not consistent over all levels of initial WLP weight loss and possibly 0-M weight (see below). Because of the importance of accounting for non-parallel covariate adjustments [43], we retained the 0-M weight interaction term even though the *p *value was slightly larger than .10. (Table 2, augmented model)

**Table 2:** ANCOVA models, and solution for final model

		Initial model			Augmented Model			Final Model			Solution in final model			
**Effect**	**Level**	**df**	**F**	**p <**	**df**	**F**	**p <**	**df**	**F**	**p <**	**Parameter Estimate**	**Std Error**	**Confidence Limits**	

	Intercept										1.65	2.98	-4.22	7.51

Treatment assignment (tx)	SS	1	2.79	0.0974	1	3.24	0.0722	1	3.84	0.0510	-8.18	4.17	-16.39	.04

Race	Non-white	1	1.02	0.3165	1	0.79	0.3754	1	1.80	0.1827	-2.03	1.51	-5.03	0.97

Sex	Male	1	0.06	0.8136	1	0.13	0.7177	1	0.00	0.9977	0.00	0.94	-1.86	1.86

Phase I weight change		1	0.55	0.4644	1	2.92	0.0903	1	2.62	0.1102	0.19	0.12	-0.04	0.42

Randomization weight		1	0.67	0.4111	1	0.09	0.7670	1	0.05	0.8248	-0.01	0.03	-0.06	0.05

Phase I weight change*tx	SS				1	3.03	0.0829	1	4.45	0.0359	-0.32	0.15	-0.61	-0.02

Randomization weight*tx	SS				1	2.59	0.1088	1	2.46	0.1176	0.06	0.04	-0.02	0.14

Education	1. High School or less							3	3.71	0.011	4.44	1.34	1.80	7.09

	2. Some College										1.32	0.86	-0.39	3.02

	3. College degree										0.69	0.92	-1.12	2.50

	5. Part college (reference)										0.00	.	.	.

Phase II %attendance	1. < 50%							4	3.62	0.006	2.30	1.33	-0.35	4.95

	2. 50-63%										4.35	1.13	2.13	6.56

	3. 75%										3.11	1.11	0.93	5.30

	4. 87.5%										2.53	1.07	0.43	4.63

	5. 100% (reference)										0.00	.	.	.

### Additional predictors of primary outcome

In the augmented model, we tested whether any of six covariates (described in Methods) or their interaction with treatment arm improved prediction of the primary outcome (weight change 12 M-(0-M)). Of these, only the main effect of two covariates, percentage attendance at weight-loss maintenance sessions and education, were significant when added singly to the augmented model, and remained significant when both were added to the augmented model to create the final model (Table 2). In the final model, the adjusted treatment effect approached significance (F[1,275] = 3.84, p < .051), but since one of the interaction terms, between treatment and weight loss in the WLP, was still significant (p < .0359), the effect of TAT vs. the SS control cannot be interpreted in terms of the main effect alone. The other interaction had a reduced effect (p < .1176) and is not considered further.

### *Post hoc *contrasts on significant interaction

**Figure 4:**
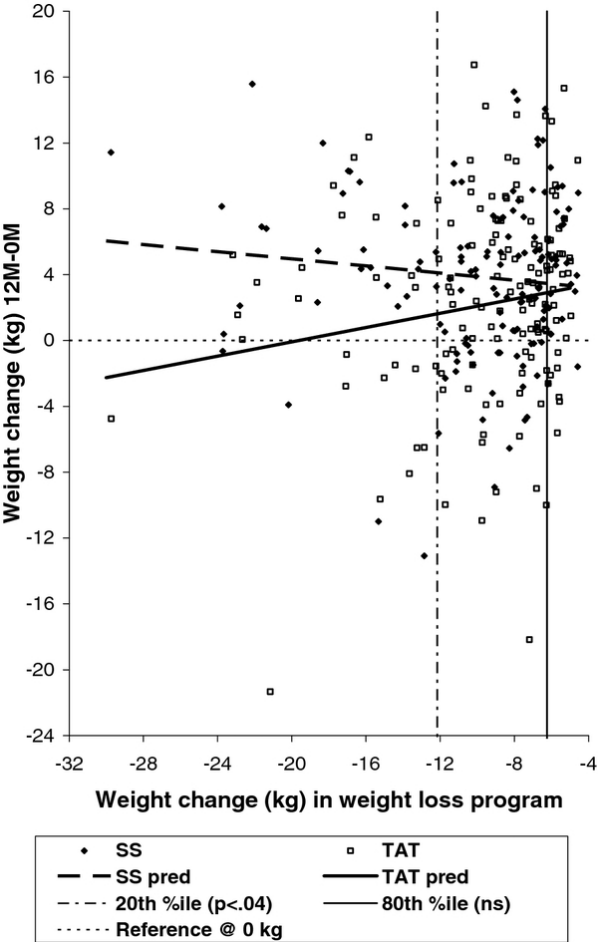
**Relationship of initial weight loss to weight regain, by treatment arm**.

We followed up the finding of a significant interaction between treatment arm and initial weight loss with further, *post hoc *analyses to estimate the TAT treatment effect at different levels of initial weight loss. A plot of the trend lines of predicted primary outcome values (Figure 4) over varying WLP weight loss in the two treatment arms, showed that TAT participants with more initial weight loss regained less weight during maintenance while SS participants with more initial weight loss regained more weight during maintenance. This suggested the *post hoc *hypothesis that the treatment effect was greatest in participants with the most WLP weight loss but negligible in participants with the smallest WLP loss (closest to the randomization criterion of -4.5 kg). We estimated and tested the difference between treatments (SS-TAT) in the least squares mean weight change when the covariate was fixed at each of eight values of its distribution in the study sample (10^th^, 15^th^, 20^th^, 25^th^, 75^th^, 80^th^, 85^th^, and 90^th ^percentiles). Weight loss was defined as a negative number, so lower percentiles represent more weight loss.

As Table 3 shows, the pattern of test results, with declining *t *and increasing *p*-value as WLP weight loss declined, was consistent with our exploratory observation. For example, the model predicts that a participant who lost at least 12.16 kg *(20^th ^percentile) *in the initial WLP would have a weight regain of 1.69 kg less if assigned to TAT than if assigned to SS (p < .0385). At the highest percentiles (i.e., WLP weight loss closest to -4.5 kg), the contrasts on least squares means were not significant.

**Table 3:** Differences in weight-loss maintenance between SS and TAT at various levels of initial weight loss

Covariate: Weight Change (kg) during weight loss program (WLP)								
**Outcome: Weight change 0-month to 12-month follow-up**								

**%ile WLP loss**	**WLP loss @ %ile (kg)**	**Predicted weight change 12M-(0M)**		**SS-TAT**	**Approx**. **s.e**.	Approx effect size*	**t**	**p <**
							
		**SS**	**TAT**					

10	-16.34	4.01	1.00	3.00	1.22	0.15	2.46	**0.0146**

15	-13.89	3.70	1.47	2.23	0.95	0.14	2.35	**0.0200**

20	-12.16	3.48	1.79	1.69	0.81	0.12	2.09	**0.0385**

25	-11.41	3.38	1.94	1.45	0.76	0.11	1.89	0.0603

75	-6.53	2.77	2.86	-0.09	0.87	-0.01	-0.10	0.9188

80	-6.24	2.73	2.91	-0.18	0.90	-0.01	-0.20	0.8393

85	-6.00	2.70	2.96	-0.26	0.92	-0.02	-0.28	0.7797

90	-5.68	2.66	3.02	-0.36	0.95	-0.02	-0.38	0.7066

*Based on estimates of LS means and approximate S.E. combined over imputations and N = 285

### Secondary outcomes

At 6 M, least square mean weight change, adjusting only for the initial planned covariates, was 0.86 kg (se 0.62) for TAT and 0.75 kg (se 0.66) for SS, with between-arm difference for six months (FV1) of 0.11 kg (se 0.52, F[1,279] = 0.048, p < .825). Similarly, no significant difference was observed between treatments among the secondary outcomes of change from 0-M to 12 M in depression, stress, insomnia, or quality of life (all p > .5, see Table 4)

**Table 4:** Between arm differences in changes in secondary psychosocial outcomes (12 M-(0-M))

Outcome measure	estimate	Std error	LCL	UCL	p <
PHQ8 (Depression)	-0.16	0.64	-1.52	1.21	0.8112

PSS (Stress)	-0.55	0.88	-2.37	1.27	0.5405

ISI (Insomnia)	-0.13	0.60	-1.38	1.12	0.8326

Q-les-Q-sf (Quality of Life)	-0.52	1.77	-4.23	3.19	0.7726

(LCL = lower confidence limit, UCL = upper confidence limit)

### Additional exploratory analyses

We obtained results similar to the primary outcome analysis above when BMI was substituted for weight in the primary outcome model. In addition, between-arm differences were not significant, in logistic regression, on three dichotomous weight-loss criteria based on weight at the 12 M follow-up: maintaining at least 4.54 kg weight loss from pre-WLP entry weight, maintaining weight less than or equal to pre WLP entry weight, and maintaining weight less than or equal to 5% below pre-WLP entry weight (all p > .7). Within both SS and TAT arms, outcomes did not vary significantly based upon practitioner assignment. Within the TAT arm, we found no significant impact of self-reported TAT practice on weight change at either 6 M or 12 M follow up.

### Adverse events

There were no unanticipated intervention related serious adverse events reported by participants.

## Discussion

Our primary analysis showed no significant difference in weight change between TAT and SS at 12 months post randomization. In addition, we observed no between group differences in changes in stress, depression, quality of life, and insomnia. Insofar as TAT, and other mind-body interventions, may be postulated to work through modulation of such factors, results suggest no difference in effect between TAT and SS for study participants.

Similarly, we found that while intervention session attendance predicted weight loss maintenance, self reported TAT home practice did not. The findings can potentially be interpreted as consistent with no effect of the TAT intervention on weight change, beyond the benefit of group support. On the other hand, practitioners of energy psychology techniques maintain that symptoms, illness, or counterproductive behavior may be caused by or attributable to suppressed trauma or emotions, and that, through proper application of the energy psychology practice, these factors can be healed, or definitively resolved. Through the lens of such a paradigm, more practice is not necessarily better. Further research is required toward clarifying the mechanism of action of TAT and other energy psychology techniques. Such an evidence based mechanistic framework will enable clinicians and investigators to better determine, prescribe, and measure the appropriate frequency of practice in future clinical trials.

In our study mean initial weight loss was 9.8 kg overall, with adjusted 12 month weight regain of 1.72 kg and 2.96 kg for TAT and SS respectively. These results are comparable with what has been reported in other long-term weight management trials. In the Weight Loss Maintenance Trial [21], for example, mean initial weight loss was 8.5 kg, while 12 month weight regain for participants in the self directed (no treatment ) arm was 3.7 kg, compared with 2.69 kg weight regain for an interactive technology arm and 2.12 kg weight regain for the personal contact arm. For both TAT and SS participants, weight regain was thus relatively modest, and considerably less than the 9.8 kg of average weight loss initially achieved.

For the secondary analysis, the final regression model included three statistically significant predictors of weight loss maintenance: session attendance, education, and the interaction between treatment assignment and initial weight loss. The strongest predictor of weight loss maintenance was attendance at weight loss maintenance group sessions. The finding is not unexpected, given the strong association between session attendance and weight loss documented in our previous trials, [32,33] and suggests the importance of the social support offered in groups, and potentially the facilitators. Similarly, the observed significant association between education and weight loss maintenance is not surprising, with better educated participants maintaining more weight loss at the 12 M visit.

The significant interaction term is of special importance. Because a significant interaction between treatment assignment and initial weight loss was found, a major assumption underlying the main effect test of treatment effect in the primary analysis model was not met, and the test on the main effect term is interpreted with caution. The significant interaction between treatment arm and amount of weight loss in the initial weight-loss program suggests a treatment effect that varied depending upon initial weight loss. Post hoc analysis was then indicated so as to identify what the TAT treatment effect was at various levels of initial weight loss. This exploratory *post hoc *analysis suggested that participants who lost the most weight in the initial weight-loss program maintained more weight loss if assigned to TAT than if assigned to SS. As Figure 4 shows, the estimated weight regain between 0-M and 12 month-follow up for SS increases with increasing weight loss in the initial weight-loss program, while the estimated weight regain between 0-M and 12-month follow up for TAT decreases with increasing weight loss in the initial weight-loss program. The direction of the association of these two variables in the SS arm is consistent with the relationship observed in data from the Weight Loss Maintenance Trial [21,44]. In the TAT arm, however, this expected pattern was reversed, with greater initial weight loss predictive of *improved *weight-loss maintenance.

Explanations for this possible conditional treatment effect are speculative but may suggest direction for future research. Weight loss in the initial WLP may serve as a marker for other participant characteristics that we either did not measure or that we cannot measure as precisely as weight. It may be that those with greater initial weight loss were more motivated, and that TAT is most appropriate for highly motivated individuals. The TAT intervention does not have obvious face validity as a strategy for weight control, which may represent a barrier to its effective use, hence requiring a higher level of enthusiasm to overcome; however, we did not measure enthusiasm or motivation in this study. The TAT method may be a behavioral tool that is most effectively used by individuals who have undergone profound physical or psychological change [45], such as substantial weight loss. Finally, it is possible that a longer follow- up time period may be needed to observe a TAT effect, with this effect first noticeable in those with higher initial weight loss.

In any case, caution is warranted in interpreting this exploratory finding, due to multiple potential confounders and other limitations. Differences in age, stress, depression, and other factors can potentially influence success in managing weight, and may have confounded results. Because participants are not blind to their treatment assignment, participant expectations can affect outcomes, yet we did not measure or analyze expectancy in our study. In addition, we were not sufficiently powered to account for nesting, or clustering, in our analyses, which may introduce additional bias. Likewise, our procedure for adding covariates to the augmented model toward arriving at the final model involves multiple tests, which could potentially introduce spurious findings.

The TAT intervention is a multimodal approach, and includes not only self acupressure, but also imagery, affirmations, drinking water, and other components. Although it was our intention to study TAT as a unified or whole system approach [46,47], it is possible that just one, or several, of the individual components of the intervention could be responsible for any effect. Finally, although Table 3 describes statistically significant model predicted differences between TAT and SS for participants at higher levels of initial weight loss, the effect sizes are small.

From a clinical standpoint, there as yet exists no real consensus on what constitutes a clinically significant reduction in weight. In terms of selected outcome measures for middle aged and older adults, 4 kg has been shown to be enough for a substantial drop in blood pressure (for borderline hypertensives) [48], while a mean weight loss of 7% is enough for an effect on the probability of borderline diabetics developing frank diabetes [49]. In addition, a mean weight loss of 5% has been shown sufficient for clinically significant improvement of mobility and quality of life in older arthritis patients [50]. These data suggest that while the net weight change for participants in both study arms may be considered to be of potential clinical importance, any potential difference between the two arms is of uncertain clinical significance.

## Conclusions

Our primary analysis showed no significant difference between TAT and SS for weight loss maintenance. The secondary analysis suggested a TAT treatment effect that was conditional on initial weight loss, while exploratory post hoc tests suggested that among participants with the highest initial weight loss, those in the TAT condition regained less weight than those in the SS condition. Multiple potential confounders and other limitations preclude firm conclusions. Further research is necessary to elucidate the effectiveness and appropriate role for TAT, and potentially other mind and body interventions, in the setting of long-term weight management.

## Competing interests

The authors declare that they have no competing interests.

## Authors' contributions

CE helped conceive and design the project, oversaw acquisition of the data, participated in coordination of the project, and drafted the manuscript. CG (Gullion) performed the statistical analysis. LD helped with analysis and interpretation of the data, and participated in coordination of the project. KF coordinated the study implementation and helped with analysis and interpretation of the data. NL helped with analysis and interpretation of the data, and participated in coordination of the project. CR helped conceive and design the project. GM helped perform the statistical analysis. CG (Gallison) helped conceive and design the project, and supervised delivery of the experimental intervention. VS helped conceive and design the project, and participated in coordination of the project. All authors read and approved the final manuscript.

## Funding

This work was funded by a grant (5R01AT003928) from The National Center for Complementary and Alternative Medicine, National Institutes of Health.

## Pre-publication history

The pre-publication history for this paper can be accessed here:

http://www.biomedcentral.com/1472-6882/12/19/prepub

## Supplementary Material

Additional file 1 **Checklist of Items for Reporting Trials of Nonpharmacologic Treatments**. Click here for file
